# Radiation‐induced AXL upregulation in CCR2^+^ monocytes promotes cardiac fibrosis

**DOI:** 10.1002/ctm2.70852

**Published:** 2026-10-13

**Authors:** Ying‐Yi Li, Jian‐Qi Xiong, Zhi‐Hui Zhang, Xin Liu, Yi‐Xin Cheng, Yong‐Xin Chi, Guan‐Qiao Li, Wen‐Huan Zhong, Shuang Chen, Zhen‐Yu He

**Affiliations:** ^1^ Department of Radiation Oncology State Key Laboratory of Oncology in South China Guangdong Provincial Clinical Research Centre for Cancer Guangdong Key Laboratory of Nasopharyngeal Carcinoma Diagnosis and Therapy Sun Yat‐sen University Cancer Centre Guangzhou China; ^2^ Department of Medical Oncology The First Affiliated Hospital Sun Yat‐sen University Guangzhou China; ^3^ Department of Medical Oncology Institute of Precision Medicine Centre for Translational Medicine The First Affiliated Hospital of Sun Yat‐sen University Guangzhou China; ^4^ Affiliated Hospital of Guangdong Medical University & Zhanjiang Key Laboratory of Zebrafish Model for Development and Disease Guangdong Medical University Zhanjiang China; ^5^ Department of Breast Surgery Hainan General Hospital (Hainan Affiliated Hospital of Medical University) Haikou China

**Keywords:** AXL, CCR2^+^ monocytes, fibroblast activation, MEK/ERK signalling, radiation‐induced cardiac fibrosis

## Abstract

**Background:**

Thoracic radiotherapy can cause delayed cardiac fibrosis, but the immune mechanisms linking radiation injury to fibroblast activation remain unclear. We investigated whether CCR2^+^ monocytes contribute to radiation‐induced cardiac fibrosis and whether AXL regulates their fibroblast‐activating activity.

**Methods and materials:**

C57BL/6 mice received 20 Gy whole‐thorax irradiation, and remodelling was assessed from IR_D0 to IR_D60. Bulk RNA sequencing was integrated with single‐cell datasets and CIBERSORTx deconvolution, lineage tracing, and tissue staining. CCR2^+^ monocyte function was examined by CCR2 neutralisation, and circulating CCR2^+^Ly6Cʰ^i^ monocytes were quantified by flow cytometry. AXL was investigated using inducible myeloid Axl deletion and bemcentinib (R428). MAPK signalling was assessed by transcriptomic analysis and immunoblotting of sorted CCR2^+^Ly6Cʰ^i^ monocytes. Conditioned medium from sorted monocytes after Axl deletion, AXL inhibition with R428, or MEK1/2 inhibition with U0126 was applied to cardiac fibroblasts.

**Results:**

Cardiac fibrosis was established by IR_D30, while leukocyte infiltration and apoptotic signals remained above baseline. Transcriptomic analyses and lineage tracing showed increased cardiac accumulation of CCR2^+^ monocytes. CCR2 neutralisation reduced circulating CCR2^+^Ly6Cʰ^i^ monocytes and attenuated interstitial collagen deposition and fibrotic marker expression. Axl was enriched in the CCR2^+^ monocyte compartment. Myeloid Axl deletion and R428 reduced cardiac fibrosis. Axl deletion suppressed MAPK‐related transcriptional programs, whereas genetic or pharmacological AXL targeting reduced MEK and ERK phosphorylation in sorted monocytes. Conditioned medium from IR_D30 monocytes induced COL1A1 and α‐SMA expression in fibroblasts, and this effect was reduced by Axl deletion, R428, or U0126 treatment of donor monocytes.

**Conclusions:**

AXL signalling contributes to the fibroblast‐activating paracrine activity of cardiac CCR2^+^ monocytes after irradiation, at least in part through MEK/ERK signalling.

**Key points:**

- Radiation exposure induces cardiac fibrosis through immunestromal interactions.
- CCR2^+^ monocytes promote fibroblast activation during radiation-induced cardiac fibrosis.
- AXL regulates monocyte‐mediated fibroblast activation through MEK/ERK signaling.
- Targeting the AXL‐MEK/ERK pathway may provide a potential strategy for preventing radiation‐induced cardiac fibrosis.

## Graphical abstract

1. IR_D30 is characterised by established cardiac fibrosis and ongoing immune remodelling.

2. Cardiac CCR2^+^ monocytes accumulate after irradiation and contribute to fibroblast activation through paracrine signalling.

3. AXL contributes to this fibroblast‐activating activity, at least in part through MEK/ERK signalling.

4. Genetic and pharmacological targeting of AXL–MEK/ERK signalling limits cardiac fibrosis and monocyte‐mediated fibroblast activation.

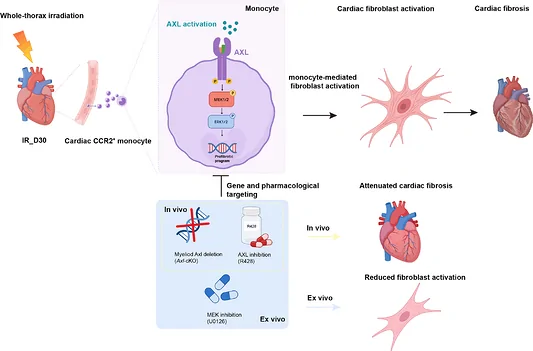

## 1 BACKGROUND

Radiotherapy is integral to the treatment of thoracic and mediastinal malignancies, but incidental cardiac exposure can produce cardiovascular toxicity that emerges years after treatment.^1^, ^2^ In breast cancer survivors, the rate of major coronary events increased by 7.4% per Gy of mean heart dose, beginning within five years of radiotherapy and remaining elevated for at least two decades.^3^ Radiation‐induced heart disease encompasses ischemic heart disease, pericardial and valvular disorders, conduction abnormalities, and cardiomyopathy.^4^, ^5^ Fibrotic remodelling is one major pathological response to cardiac irradiation. Progressive interstitial matrix deposition increases myocardial stiffness and disrupts cardiac structure and function,^6^, ^7^ yet the cellular processes that connect radiation injury to sustained fibrosis remain incompletely understood.

Activated fibroblasts are the principal matrix‐producing cells in cardiac fibrosis, but their behaviour is shaped by signals from injured parenchymal cells, vascular cells and the immune compartment.^8^, ^9^ T and B lymphocytes, neutrophils, resident macrophages, and infiltrating monocytes can each influence fibroblast activation, with their contributions varying according to the cause and stage of injury.^10^, ^11^ Recent work in human heart failure identified spatially organised interactions between CCR2^+^ monocytes/macrophages and disease‐associated fibroblasts.^12^ Single‐cell studies have also begun to define radiation‐associated changes in cardiac macrophage states.^13^ In a model combining radiotherapy with immunotherapy, extensive immune–fibroblast communication was linked to an interleukin (IL)‐6‐centred circuit.^14^ However, whether specific monocyte populations contribute functionally to fibrosis after irradiation alone, particularly during the transition from early injury to established fibrotic remodelling, remains unclear.

CCR2^+^ monocytes are plausible candidates for mediating this transition. Signalling through the CCL2–CCR2 axis controls the mobilisation and tissue entry of inflammatory monocytes, which can differentiate into macrophages and release mediators that alter neighbouring stromal cells.^15^, ^16^ In myocardial infarction and other acute cardiac injuries, these cells participate in inflammatory clearance and scar formation, but prolonged or dysregulated monocyte activity can also promote adverse remodeling.^17^, ^18^ Reparative scar formation after acute ischemic injury is not equivalent to the progressive interstitial fibrosis that follows cardiac irradiation. It therefore remains necessary to determine whether CCR2^+^ monocytes contribute functionally to radiation‐induced cardiac fibrosis rather than merely accumulating as part of the inflammatory response.

AXL, a member of the TYRO3–AXL–MERTK receptor tyrosine kinase family, regulates cell survival, efferocytosis, migration, and inflammatory adaptation in response to tissue injury.^19^, ^20^ GAS6–AXL signalling has been implicated in fibrotic remodelling in the liver, lung, and other organs, with reported activity in hepatic stellate cells, fibroblasts, endothelial cells, and myeloid populations.^21^, ^22^ In the heart, myocardial AXL expression and circulating soluble AXL are elevated in patients with advanced heart failure, while experimental pressure overload induces *Axl* during the early stages of ventricular remodelling.^23^, ^24^ Myeloid AXL can also promote maladaptive inflammation after myocardial ischemia–reperfusion injury.^25^ Recent studies in pulmonary fibrosis further suggest that AXL can regulate profibrotic programs in Ly6C^hi^ monocytes,^22^ although whether a similar mechanism operates in the irradiated heart remains unknown. These studies place AXL at the intersection of fibrotic and inflammatory remodelling, while showing that its function is not restricted to monocytes. The unresolved question is whether AXL is induced and functionally active in CCR2^+^ monocytes during cardiac remodelling after irradiation, and whether it regulates the ability of these cells to promote fibroblast activation.

Here, we investigated how inflammatory myeloid responses are coupled to the development of cardiac fibrosis after whole‐thorax irradiation. We first defined the temporal and cellular changes accompanying the transition from injury to fibrosis, and then used lineage tracing and CCR2 neutralisation to examine the involvement of CCR2^+^ monocytes. Integrated bulk and single‐cell transcriptomic analyses subsequently prioritised *Axl* as a candidate regulator of the later remodelling stage. We assessed its function using inducible myeloid *Axl* deletion and pharmacological AXL inhibition. Signalling analyses in sorted cardiac monocytes and conditioned‐medium experiments with primary cardiac fibroblasts were then used to examine the downstream mechanism. The findings support a model in which AXL signalling in CCR2^+^ monocytes contributes to their fibroblast‐activating paracrine activity, at least in part through MEK/ERK signalling.

## 2 METHODS AND MATERIALS

### 2.1 Mouse models

Lyz2^CreERT2^ (strain T052789) and Axl^fl/fl^ (strain T009234) mice were obtained from GemPharmatech, and Rosa26^tdTomato^ mice were obtained from The Jackson Laboratory. Mice of both sexes were enrolled at 6–8 weeks of age and randomly allocated to the indicated experimental groups. Animals were maintained under specific‐pathogen‐free conditions with a 12‐h light/dark schedule and unrestricted access to food and water. All animal procedures were conducted in accordance with protocols approved by the Institutional Animal Care and Use Committee. Lyz2^CreERT2^; Rosa26^tdTomato^ and Lyz2^CreERT2^; Axl^fl/fl^; Rosa26^tdTomato^ mice received tamoxifen intraperitoneally (Sigma, T5648‐1G; 0.08 mg/g body weight) once daily for 5 consecutive days to induce Cre‐mediated recombination before subsequent experiments.

### 2.2 Mouse radiation experiments

C57BL/6J, Lyz2^CreERT2^; Axl^fl/fl^; Rosa26^tdTomato^ and Lyz2^CreERT2^; Rosa26^tdTomato^ mice received a single 20 Gy dose of whole‐thorax radiation (165 kV, 25 mA X‐rays; Rs2000, USA). Cardiac tissues were collected at the designated experimental endpoints according to the requirements of each analysis.

### 2.3 Anti‐CCR2 antibody treatment

To reduce circulating CCR2^+^ monocytes, irradiated C57BL/6J mice received an anti‐mouse CCR2 monoclonal antibody (AbAb‐CCR2, mouse IgG2a Fc Silent; Absolute Antibody, Ab02444‐2.3). Mice were administered 20 µg of antibody by intraperitoneal injection once daily from IR_D3 to IR_D6. Control mice received an equal dose of a mouse IgG2a Fc‐silent isotype control antibody (matched control supplied by Absolute Antibody) following the same schedule. Peripheral blood was collected from the tail vein on IR_D7 for flow cytometric analysis of circulating monocytes. For peripheral blood analysis, blood samples were subjected to red blood cell lysis, washed, and stained using the same antibody panel. At IR_D30, mice were perfused with phosphate‐buffered saline, and hearts were collected for histological analyses.

### 2.4 Histological assessment of cardiac fibrosis

Residual blood was cleared by perfusing mice with ice‐cold phosphate‐buffered saline (PBS) before heart excision. The excised hearts remained in 4% paraformaldehyde at room temperature for 24 h, then underwent dehydration and paraffin processing before sectioning at 5 µm. Myocardial morphology was examined on haematoxylin and eosin (H&E)‐stained sections (Solarbio, G1120). Collagen deposition was assessed with Masson's trichrome (Solarbio, G1340) and Sirius Red (Solarbio, G1472). Interstitial and perivascular fibrosis were quantified separately. For interstitial fibrosis, large vessels, vascular walls, and surrounding perivascular regions were excluded from the regions of interest. Perivascular fibrosis was assessed independently using vessel‐centred regions of interest. Images were analysed with ImageJ/Fiji using predefined colour‐thresholding criteria that were applied consistently across all groups. Fibrosis was expressed as the percentage of positively stained area within the corresponding region of interest.

### 2.5 TUNEL staining

Paraffin‐embedded cardiac sections prepared as described above were used for TUNEL labelling with the One Step TUNEL Apoptosis Assay Kit (green fluorescence; Beyotime, C1086). After deparaffinization and rehydration, we permeabilised the sections with proteinase K and rinsed them in PBS. Labelling with the TUNEL reaction mixture proceeded for 60 min at 37°C, with the sections protected from light. DAPI counterstaining was followed by fluorescence imaging, with acquisition settings held constant within each experiment. In ImageJ/Fiji, we calculated the percentage of all DAPI‐positive nuclei that were also TUNEL‐positive.

### 2.6 Immunohistochemistry and multiplex immunohistochemistry

Paraffin sections prepared as described above were used for immunohistochemical staining. After dewaxing and rehydration, endogenous peroxidase activity was quenched with 3% H_2_O_2_. Antigen retrieval was then performed by heating the sections in an EDTA‐based buffer. Sections were incubated at 4°C overnight with primary antibodies against α‐SMA (1:300, CST, 19245T), Collagen I (1:250, Abcam, AB270993), CD45 (1:500, Abcam, ab10558), cleaved caspase‐3 (1:600, CST, 9664T), CCR2 (1:300, Abcam, AB273050), AXL (1:300, CST, 8661T), RFP (1:800, Rockland, 600‐401‐379), and GAS6 (1:200, Affinity, DF8659). After removal of unbound primary antibody by PBS washing, sections were exposed to the corresponding HRP‐conjugated secondary antibodies for 30 min at 37°C. Immunoreactivity was visualised using DAB, followed by haematoxylin counterstaining.

For multiplex immunohistochemistry, tissue sections underwent the same initial deparaffinization and antigen‐retrieval procedures. After primary‐antibody incubation, sections were treated with the secondary detection reagent (PPA200) for 10 min at room temperature. Fluorescent signal development was performed with PPD520 or PPD570 for 10 min, followed by washing with TBST. Nuclear counterstaining was carried out with DAPI (1:1000) for 1 min.

Stained sections were imaged using a TissueFAXS tissue cytometry imaging system (TissueGnostics, Austria) with a 20× objective under identical acquisition settings within each experiment. Quantitative analysis was performed in predefined myocardial regions using identical analysis criteria across groups. Quantification of Collagen I and α‐SMA was restricted to myocardial interstitial regions, with large vessels and perivascular regions excluded. Positive staining was quantified in ImageJ/Fiji and reported as the fraction of the analysed interstitial area occupied by the corresponding signal.

### 2.7 Flow cytometry sorting of CCR2^+^Ly6C^hi^ monocytes

Hearts collected after PBS perfusion were briefly washed in cold PBS and mechanically minced before enzymatic dissociation. Tissue fragments were incubated for 30 min at 37°C with gentle agitation in PBS containing 2 mM CaCl_2_, 0.5% bovine serum albumin (BSA), collagenase II (1 mg/mL, Stemcell, 07418), and DNase I (1 mg/mL, Stemcell, 07902).

After digestion, the cell suspension was passed through a 40‐µm strainer and pelleted by centrifugation at 300 × g for 5 min. Residual erythrocytes were removed with red blood cell lysis buffer (Biosharp, BL503A) when required. Cells were subsequently washed and transferred into staining buffer (BD Biosciences, 554657).

For surface‐marker staining, the cell suspension was incubated at 4°C for 30 min with CD45‐PE‐Cy7 (1:500, TONBO, 60‐0451‐U100), CD11b‐PE (1:300, BioLegend, 101208), Ly6G‐APC‐Cy7 (1:300, BioLegend, 127623), Ly6C‐PerCP‐Cy5.5 (1:300, Invitrogen, 45‐5932‐80) and CCR2‐FITC (1:300, BioLegend, 150608). Nonviable cells were identified using Ghost Dye™ Red 710 (1:500, TONBO Biosciences) and excluded from subsequent analysis.

CCR2^+^Ly6C^hi^ monocytes were sorted using a FACSAria cell sorter (BD Biosciences). The sequential gating strategy was FSC/SSC → singlets → live cells → CD45^+^ → CD11b^+^ → Ly6G^−^ → Ly6C^hi^ → CCR2^+^.

### 2.8 Primary cardiac fibroblast isolation

The cardiac cell suspension was prepared by the enzymatic digestion described above. A 70‐µm strainer removed residual tissue fragments, after which cells were recovered by centrifugation at 300 × *g* for 5 min and resuspended in complete culture medium.

Preferential adhesion was used to enrich fibroblasts. The mixed suspension was plated for 60–90 min at 37°C, allowing fibroblasts to attach rapidly. Gentle PBS washing then removed most cells that had not attached. The adherent population was maintained at 37°C under 5% CO_2_ in Dulbecco's modified Eagle medium (DMEM; Gibco) supplemented with 10% foetal bovine serum and 1% penicillin–streptomycin and was subsequently used for conditioned‐medium stimulation.

### 2.9 Pharmacological intervention and signalling validation

To inhibit AXL in vivo, C57BL/6J mice in the experimental group received bemcentinib (R428/BGB324; 25 mg/kg; Selleck Chemicals, S2841) dissolved in 1% sodium carboxymethylcellulose (CMC‐Na) by oral gavage once daily from IR_D7 to IR_D30 post‐irradiation. Control animals were administered the corresponding vehicle. Heart tissues were collected at IR_D30 for subsequent analyses.

For ex vivo mechanistic validation, CCR2^+^Ly6C^hi^ monocytes were freshly isolated from irradiated hearts by fluorescence‐activated cell sorting. Cells were treated with R428 (1 µM, 24 h) or U0126 (10 µM, 6 h; MEK1/2 inhibitor; Selleck Chemicals, Cat# S1102) under standard culture conditions. After treatment, cells were washed twice with PBS and collected for Western blot analysis. AXL pathway activity was evaluated by measuring pAXL, AXL, pMEK1/2, MEK1/2, pERK1/2 and ERK1/2.

### 2.10 Conditioned medium and fibroblast stimulation

To evaluate paracrine effects of monocyte‐derived signals, conditioned medium (CM) experiments were performed. Conditioned medium was generated from flow‐sorted CCR2^+^Ly6C^hi^ monocytes obtained from the indicated experimental groups, including Sham and IR_D30 mice, Control and Axl‐cKO mice, or from irradiated monocytes treated ex vivo with vehicle, R428, or U0126, as appropriate for each experiment. For each donor condition, the sorted CCR2^+^Ly6Cʰ^i^ monocytes underwent two PBS washes before a 24‐h period in fresh culture medium. We harvested this medium and removed cellular debris by centrifugation at 300 × *g* for 5 min.

Primary cardiac fibroblasts were exposed to monocyte‐derived CM for 48 h. After stimulation, fibroblasts were harvested for protein extraction and western blot analysis of fibrosis‐related markers, including α‐SMA and COL1A1.

### 2.11 Western blotting

Western blot analyses were performed across four experimental systems: (i) CCR2^+^Ly6C^hi^ monocytes isolated from irradiated mouse hearts by fluorescence‐activated cell sorting; (ii) ex vivo–treated CCR2^+^Ly6C^hi^ monocytes following pharmacological inhibition with bemcentinib (R428) or U0126; (iii) cardiac fibroblasts stimulated with monocyte‐CM; and (iv) whole‐heart tissue samples.

For sorted monocytes, due to limited cell yield, samples from 2–3 mice were pooled to obtain sufficient protein for one biological replicate. Three independently prepared pooled replicates were analysed for each condition (*n* = 3). Cardiac fibroblast and tissue samples were derived from independent biological replicates without pooling unless otherwise specified.

Cells and cardiac tissue were lysed in RIPA buffer supplemented with inhibitors of proteases and phosphatases. BCA measurements were used to equalise the amount of protein loaded onto 10% sodium dodecyl sulphate–polyacrylamide gels. Following electrophoresis, proteins were transferred to PVDF membranes. Blocking used 5% non‐fat milk, with 5% BSA substituted for phosphoprotein detection. Membranes were probed overnight at 4°C with the primary antibodies listed below, then for 1 h at room temperature with the matching HRP‐conjugated secondary antibodies. Enhanced chemiluminescence was recorded on a Tanon 5200 imaging system.

Primary antibodies included pAXL (1:1000, CST, 5724T), AXL (1:1000, CST, 8661T), pERK1/2 (1:1000, CST, 9106T), ERK1/2 (1:1000, CST, 9102S), pMEK1/2 (1:1000, CST, 9121S), MEK1/2 (1:1000, CST, 9122T), GAS6 (1:1000, Affinity, DF8659), α‐SMA (1:1000, CST, 19245T), COL1A1 (1:1000, CST, 72026T) and GAPDH (1:2000, CST, 2118S). GAPDH was used as the loading control. Band densitometry was performed in ImageJ/Fiji. Phosphorylated AXL, MEK1/2, and ERK1/2 were quantified relative to their respective total‐protein signals, whereas total proteins and fibrosis‐related markers were normalised to GAPDH.

### 2.12 Quantitative real‐time polymerase chain reaction

Total RNA extracted from cardiac tissue with the RNeasy Micro Kit (Qiagen) was reverse‐transcribed using the PrimeScript RT Kit (Takara). We quantified *Acta2*, *Col1a1*, and *Col3a1* transcripts by SYBR Green detection on a CFX96 Real‐Time Polymerase Chain Reaction (PCR) System (Bio‐Rad), with three technical replicates per biological sample. Relative transcript levels were calculated using the 2^−ΔΔCt^ method, with *Gapdh* as the reference gene. Table 1 lists the primer sequences.

**TABLE 1:** Primer sequences used for quantitative polymerase chain reaction (qPCR).

Genes	Forward primer (5′–3′)	Reverse primer (5′–3′)
*Acta2*	GTCCCAGACATCAGGGAGTAA	TCGGATACTTCAGCGTCAGGA
*Col1a1*	AAGAAGCACGTCTGGTTTGG	GGTCAGCTGGATAGCGACAT
*Col3a1*	CTGTAACATGGAAACTGGGGAAA	CCATAGCTGAACTGAAAACCACC
*Gapdh*	AACGGATTTGGTCGTATTGGG	CCTGGAAGATGGTGATGGGAT

### 2.13 Data collection and processing for RNA sequencing

Cardiac RNA sequencing was performed for two independent experimental comparisons. The temporal dataset included hearts collected from C57BL/6J mice at IR_D0, IR_D7, and IR_D30 (*n* = 3 per group), whereas the genetic dataset comprised irradiated Axl‐conditional knockout mice (Lyz2^CreERT2^; Axl^fl/fl^) and their corresponding irradiated controls (*n* = 3 per group). Samples belonging to the same comparison were subjected to RNA extraction, library preparation, and sequencing within a single experimental batch.

We extracted total cardiac RNA with TRIzol reagent (Invitrogen, 15596026) and removed residual genomic DNA by treatment with RNase‐free DNase I (Invitrogen, 18068‐015). Library preparation required an RNA integrity number (RIN) of at least 7.0, as assessed on an Agilent 2100 Bioanalyzer. Novogene (Beijing, China) constructed strand‐specific mRNA libraries using the Illumina TruSeq Stranded mRNA workflow; the resulting libraries were sequenced in 150‐bp paired‐end mode on an Illumina NovaSeq 6000.

Raw‐read quality was checked with FastQC (v0.11.8) before Trim Galore (v0.6.1) was used to trim adapters and low‐quality bases. STAR (v2.7.10a) aligned the processed reads to the mouse reference genome mm10 (GRCm38),^26^ and HTSeq (v2.0.2) generated gene‐level counts.^27^

Raw gene counts were analysed in DESeq2 (v1.40.1).^28^ We obtained gene‐wise P values from the Wald test and adjusted them with the Benjamini–Hochberg procedure. For identification of genes associated with the IR_D30 remodelling stage, upregulated genes were defined by a log2 fold change ≥ 1 together with an adjusted *p*‐value < 0.05. Within each comparison, genes meeting these criteria were ranked according to adjusted P value, and the 10 most significant genes were retained. Candidate genes shared by the IR_D30‐versus‐IR_D0 and IR_D30‐versus‐IR_D7 comparisons were then identified by intersection analysis.

Pathway‐level changes were assessed by Kyoto Encyclopaedia of Genes and Genomes (KEGG) enrichment analysis and gene set enrichment analysis (GSEA) using clusterProfiler in R.^29^, ^30^ For GSEA, genes were ranked according to the DESeq2 Wald statistic, and KEGG gene sets containing 10–500 genes were analysed. Enrichment was summarised by the normalised enrichment score (NES), with significance evaluated using Benjamini–Hochberg‐adjusted *p*‐values. The MAPK signalling pathway was further examined based on its GSEA enrichment profile.

### 2.14 Processing and clustering of single‐cell RNA sequencing data

Single‐cell RNA sequencing (scRNA‐seq) data of 26,855 immune cells from radiation‐induced cardiac fibrosis (RICF) tissues were obtained from the Sequence Read Archive (SRA) database under accession number PRJNA1088304 (https://www.ncbi.nlm.nih.gov/sra/PRJNA1088304), comprising samples collected at day 0 (d0), day 7 (d7), and day 35 (d35) post‐irradiation.^13^ An additional publicly available single‐cell/single‐nucleus RNA‐seq dataset generated from an AngII‐induced cardiac remodelling model (ArrayExpress accession: E‐MTAB‐8810; https://www.ebi.ac.uk/arrayexpress/experiments/E‐MTAB‐8810/) was included as a reference dataset encompassing major cardiac cell populations, including immune, stromal, and endothelial cells and cardiomyocyte‐derived nuclei.^31^

We used Seurat (v5.0.1) in R (v4.4.1) for single‐cell analyses.^32^ Cells were retained only if they had 200–6,000 detected genes and a mitochondrial transcript fraction no greater than 10%; potential doublets were also excluded. Following this filtering, Seurat's LogNormalize procedure was applied with a scale factor of 10,000. Highly variable features were identified, and the normalised data were scaled before principal component analysis (PCA). Inspection of the ElbowPlot determined which principal components were carried forward.

The retained components provided the basis for constructing cellular neighbourhoods and shared nearest‐neighbour clustering at a resolution of 0.5. Uniform manifold approximation and projection displayed the clusters in two dimensions. We then assigned cell identities by considering both each cluster's overall transcriptional profile and established lineage‐associated markers.

### 2.15 Cell‐type deconvolution analysis

Cell‐type composition of whole‐heart bulk RNA‐seq samples was estimated using the CIBERSORTx web platform (https://cibersortx.stanford.edu/) as previously described by Newman et al.^33^ A custom cardiac cell‐type signature matrix was generated using the CIBERSORTx signature‐matrix workflow from the annotated E‐MTAB‐8810 whole‐heart single‐cell transcriptomic dataset. The reference included major cardiac cell populations, including cardiomyocytes, fibroblasts, vascular and lymphatic endothelial cells, pericytes, smooth muscle cells, mesothelial cells, Schwann cells, and major immune‐cell populations. The TPM‐normalized bulk RNA‐seq expression matrix from the IR_D0, IR_D7 and IR_D30 groups was used as the mixture matrix. Cell fractions were estimated using the CIBERSORTx Fractions module in relative mode with 100 permutations, with quantile normalisation disabled. The resulting values were interpreted as estimated relative cell proportions and were used to characterise overall changes in cardiac cellular composition across the three time points.

### 2.16 Statistical analysis

Group comparisons were performed in GraphPad Prism 10 (GraphPad Software, San Diego, CA, USA). We used unpaired, two‐tailed Student's t‐tests for two independent groups and one‐way analysis of variance (ANOVA) with Tukey's multiple‐comparison procedure for three or more groups. A *p*‐value below 0.05 was defined as statistical significance. Unless otherwise specified, n denotes the number of independent biological replicates. Data are summarised as means with the standard error of the mean (SEM), except where the figure legends specify another display.

## 3 RESULTS

### 3.1 Radiation establishes a transitional cardiac microenvironment with persistent inflammation and fibrosis

To trace how the cardiac microenvironment evolves after irradiation, we examined hearts at day 7 (IR_D7), day 30 (IR_D30), and day 60 (IR_D60) following whole‐thorax irradiation, with unirradiated mice serving as Sham controls (Figure 1A). Masson's trichrome and Sirius Red staining showed limited interstitial collagen deposition in IR_D7 hearts, followed by a pronounced increase at IR_D30 that persisted at IR_D60 (Figure 1B,E,F). Collagen I and α‐SMA staining followed the same temporal pattern (Figure 1C,D,G,H). Across all four fibrotic readouts, staining was markedly increased in IR_D30 compared with IR_D7, with no further significant increase in IR_D60. Perivascular collagen deposition showed a similar time course, with increased Masson's trichrome and Sirius Red staining at IR_D30 that remained elevated at IR_D60 (Figure S1A–D). Cardiac fibrosis was therefore evident in both interstitial and perivascular regions by IR_D30.

**FIGURE 1:**
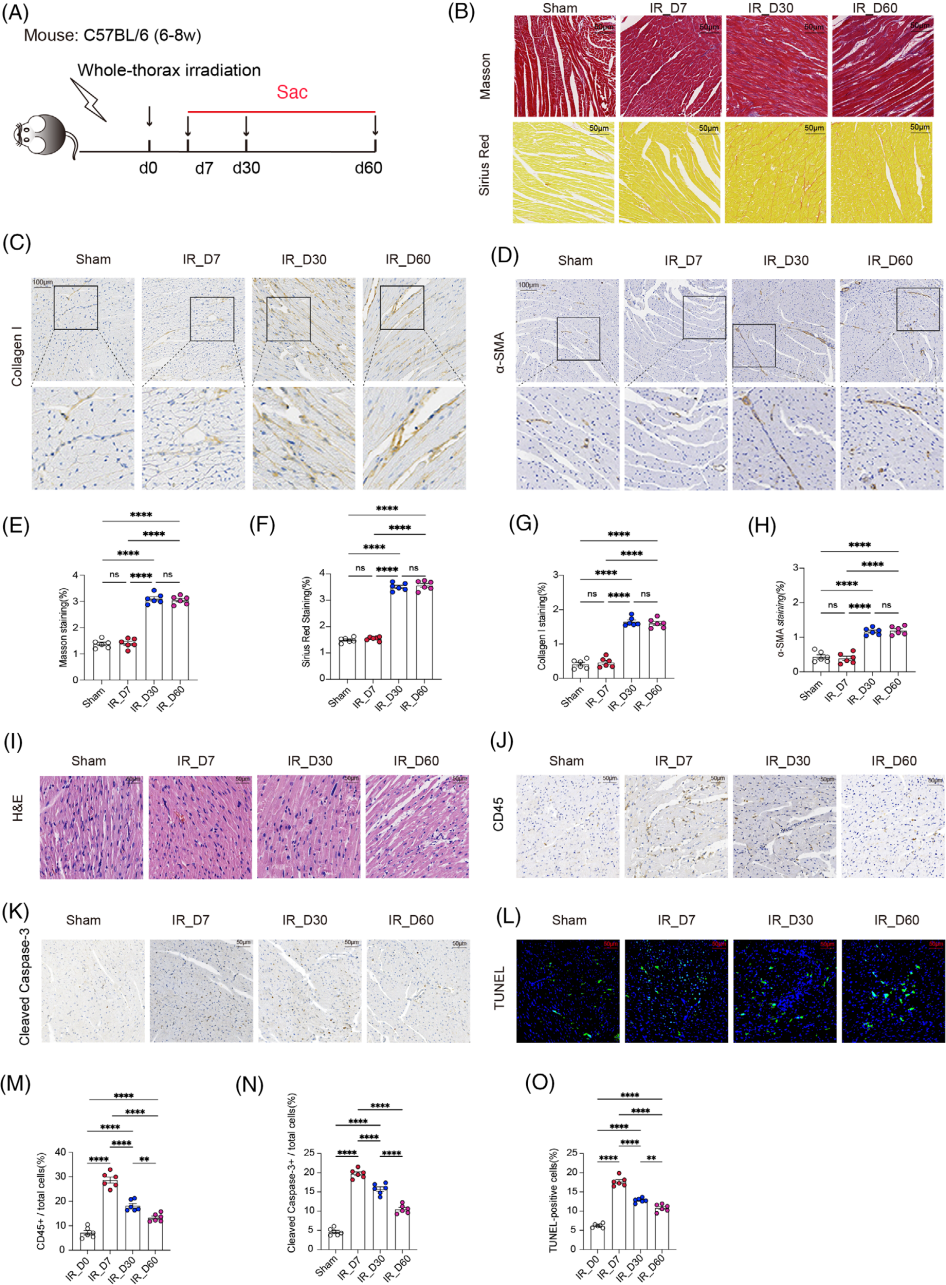
Temporal progression of cardiac injury and fibrosis following whole‐thorax irradiation. (A) Schematic diagram of the experimental design and tissue collection from Sham mice and irradiated mice at IR_D7, IR_D30, and IR_D60 after whole‐thorax irradiation. (B) Representative Masson's trichrome and Sirius Red staining of cardiac tissues from the Sham, IR_D7, IR_D30, and IR_D60 groups. Scale bar, 50 µm. (C) Representative immunohistochemical staining of Collagen I in cardiac tissues at the indicated time points. Boxed regions in the upper panels are shown at higher magnification below. Scale bar, 100 µm in the upper panels. (D) Representative immunohistochemical staining of α‐SMA in cardiac tissues at the indicated time points. Boxed regions in the upper panels are shown at higher magnification below. Scale bar, 100 µm in the upper panels. (E–H) Quantification of the Masson's trichrome‐positive area (E), Sirius Red‐positive area (F), Collagen I‐positive area (G), and α‐SMA‐positive area (H), expressed as percentages of the analyzed myocardial interstitial area. (I) Representative H&E staining of cardiac tissues from the Sham, IR_D7, IR_D30, and IR_D60 groups. Scale bar, 50 µm. (J, M) Representative immunohistochemical staining (J) and quantification of the proportion of CD45‐positive cells (M) in cardiac tissues. Scale bar, 50 µm. (K, N) Representative immunohistochemical staining (K) and quantification of the proportion of Cleaved Caspase‐3‐positive cells (N) in cardiac tissues. Scale bar, 50 µm. (L, O) Representative TUNEL staining (L) and quantification of the proportion of TUNEL‐positive nuclei among DAPI‐positive nuclei (O) in cardiac tissues. Green, TUNEL‐positive nuclei; blue, DAPI. Scale bar, 50 µm. Data are presented as mean ± SEM. n = 6 mice per group. Statistical significance was assessed using one‐way ANOVA followed by Tukey's multiple‐comparisons test. ns, not significant; **p < 0.01; ****p <0.0001.

Inflammation and tissue injury followed a related but distinct course. H&E staining revealed prominent inflammatory infiltration and disruption of myocardial architecture in IR_D7 hearts (Figure 1I). These acute changes had partially subsided in IR_D30, although inflammatory infiltrates and myocardial abnormalities remained evident. Consistent with these histological findings, the proportion of CD45‐positive cells remained significantly higher at IR_D30 than in Sham hearts, despite a reduction from IR_D7 (Figure 1J,M). In parallel, cleaved caspase‐3 and TUNEL staining showed an early peak in positivity at IR_D7, followed by a decline at IR_D30 (Figure 1K,L,N,O). These inflammatory and injury‐related changes subsided further at IR_D60, although all three readouts remained above Sham levels. IR_D30 therefore marked a transitional stage in which persistent inflammation coincided with established cardiac fibrosis. This temporal overlap prompted us to investigate the inflammatory cell populations present at IR_D30 and their potential involvement in fibrotic remodelling.

### 3.2 CCR2^+^ monocytes accumulate during radiation‐induced cardiac fibrotic remodelling

To identify the cell populations associated with the inflammatory and fibrotic microenvironment at IR_D30, we performed bulk RNA sequencing of cardiac tissues from IR_D0, IR_D7 and IR_D30 mice, with three independent biological samples in each group (Figure 2A). Cell composition was estimated by CIBERSORTx using a whole‐heart single‐cell and single‐nucleus RNA‐seq dataset (E‐MTAB‐8810) as the reference. Major cardiac populations, including cardiomyocytes, fibroblasts, endothelial cells, pericytes, smooth muscle cells, and immune cells, were annotated using representative marker genes (Figure S2A,B).

**FIGURE 2:**
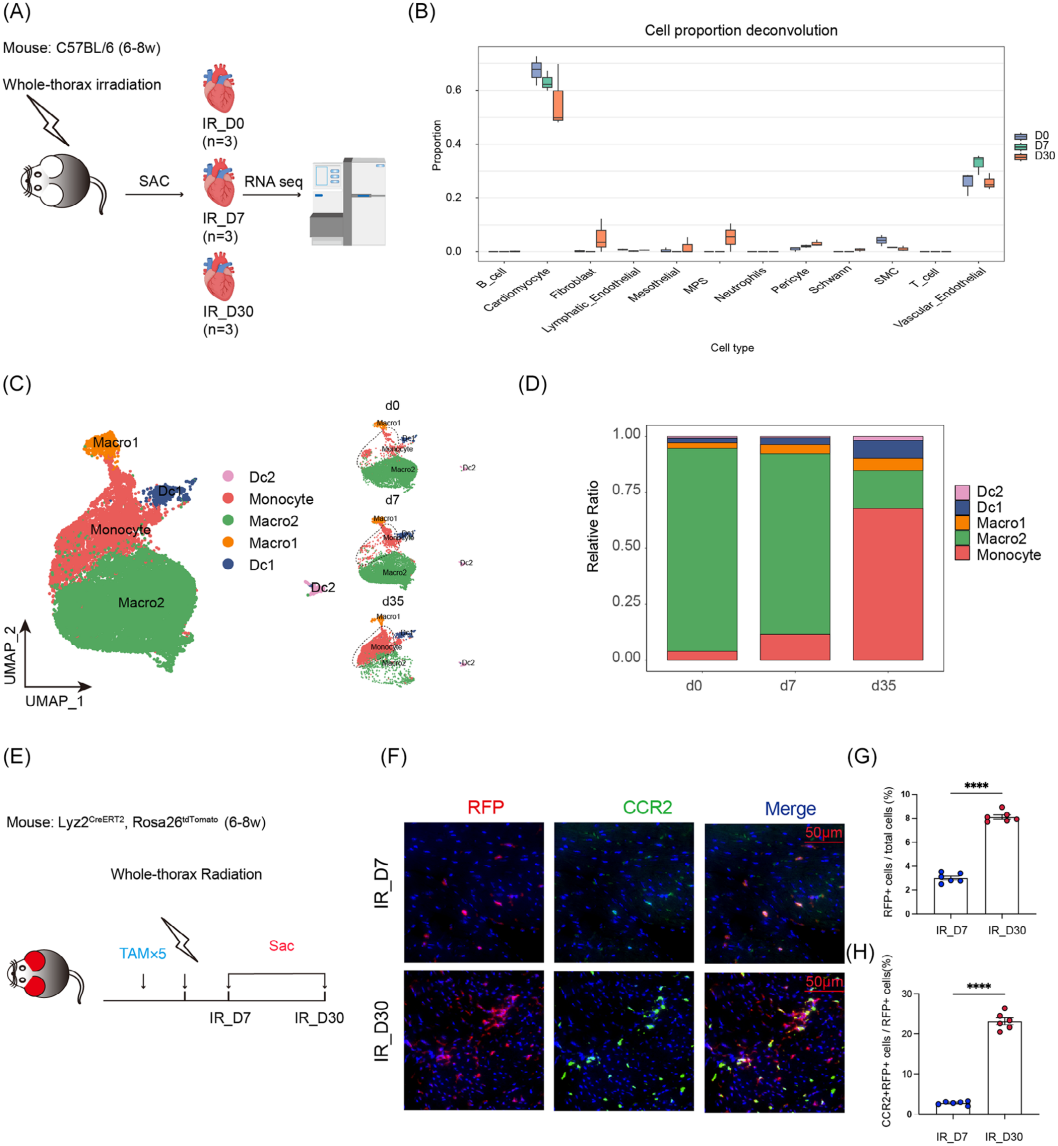
Transcriptomic and single‐cell analyses reveal increased cardiac monocyte accumulation following irradiation. (A) Schematic of the bulk RNA‐sequencing experiment using cardiac tissues collected at baseline (IR_D0) and at IR_D7 and IR_D30 after irradiation (n = 3 independent biological samples per group). (B) Cell‐proportion deconvolution of bulk cardiac RNA‐sequencing data from the IR_D0, IR_D7, and IR_D30 groups. (C) UMAP visualization of monocyte, macrophage, and dendritic‐cell subsets identified in the public radiation‐associated cardiac single‐cell RNA‐sequencing dataset, with their distributions at d0, d7, and d35 shown on the right. (D) Relative proportions of Dc2, Dc1, Macro1, Macro2, and Monocyte populations at d0, d7, and d35. (E) Schematic of lineage tracing in Lyz2^CreERT2^; Rosa26^tdTomato^ mice following whole‐thorax irradiation. (F) Representative immunofluorescence staining of RFP and CCR2 in cardiac tissues at IR_D7 and IR_D30. Scale bar, 50 µm. (G) Quantification of RFP‐positive cells among total cardiac cells. (H) Quantification of CCR2^+^RFP^+^ cells among RFP^+^ cells. Data in panels G and H are presented as mean ± SEM. n = 6 mice per group. Statistical significance was assessed using an unpaired two‐tailed Student's t‐test. ****p < 0.0001.

Within the stromal compartment, the estimated proportion of fibroblasts increased at IR_D30. This change was consistent with the cardiac fibrosis observed in our irradiation model. Within the immune compartment, the mononuclear phagocyte system (MPS) showed the most pronounced increase at IR_D30. By contrast, the estimated proportions of T cells, B cells, and neutrophils remained largely unchanged (Figure 2B). Because cardiac inflammation remained above baseline at IR_D30, the selective increase in MPS led us to examine this immune compartment more closely.

To determine which cell population contributed to the MPS change, we analysed radiation‐associated cardiac scRNA‐seq data collected at d0, d7 and d35. The major cardiac immune populations were annotated as MPS, NKT cells, neutrophils, and B cells according to their representative marker genes (Figure S3A,B). Further analysis resolved MPS into five populations: Dc1, Dc2, Macro1, Macro2, and monocytes (Figure 2C). The monocyte population was distinguished by the combined expression of several monocyte‐associated genes, including *Ly6c2*, *Ms4a4c*, *Lgals3* and *Ccr2* (Figure S3C). This marker profile therefore defined the population as CCR2‐positive monocytes. Their relative abundance within the MPS compartment increased markedly at d35 compared with d0 and d7 (Figure 2D), identifying CCR2‐positive monocytes as the main MPS population associated with the later remodelling phase.

We then examined whether this population also accumulated at IR_D30 in our irradiation *model*. Lyz2^CreERT2^; Rosa26^tdTomato^ mice were used to trace myeloid‐lineage cells following irradiation (Figure 2E). Immunofluorescence staining showed that the proportion of RFP‐positive cells among total cardiac cells was significantly higher at IR_D30 than at IR_D7 (Figure 2F, G). Within the RFP‐positive population, the proportion of CCR2^+^RFP^+^ cells was also increased at IR_D30 (Figure 2F, H). Together, the single‐cell and tissue‐level analyses identify CCR2‐positive monocyte accumulation as a prominent feature of the IR_D30 remodelling phase.

### 3.3 CCR2 neutralisation attenuates radiation‐induced cardiac fibrosis

To determine whether CCR2^+^ monocytes contribute to radiation‐induced cardiac fibrosis, mice were treated with a CCR2‐neutralizing antibody following whole‐thorax irradiation, with irradiated mice receiving an isotype antibody serving as controls (Figure 3A). Peripheral blood flow cytometry at IR_D7 showed a marked reduction in circulating CCR2^+^Ly6C^hi^ monocytes after anti‐CCR2 treatment compared with isotype controls (Figure 3B, C).

**FIGURE 3:**
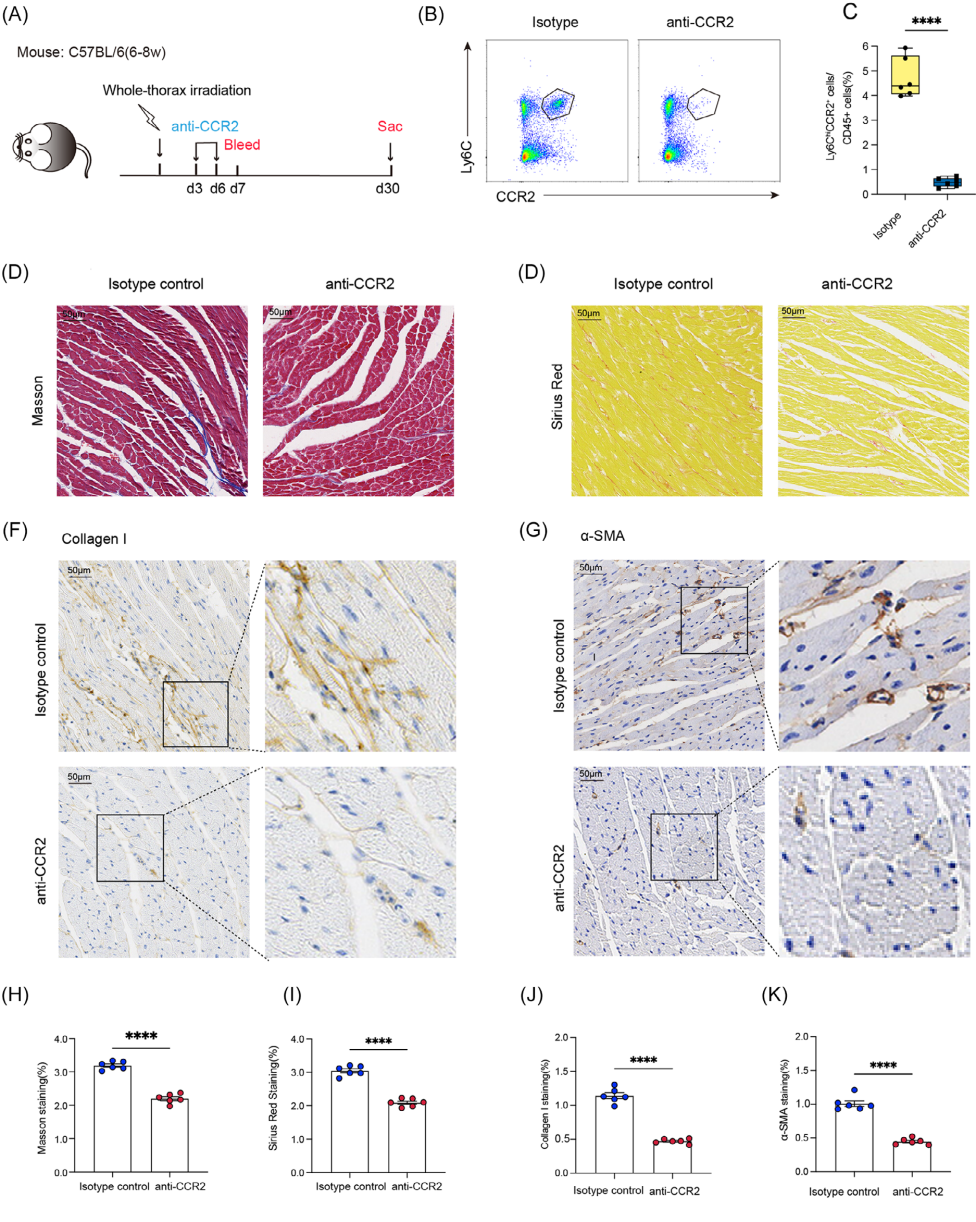
Anti‐CCR2 treatment attenuates radiation‐induced cardiac fibrosis. (A) Schematic diagram of the anti‐CCR2 treatment schedule following whole‐thorax irradiation. Mice received anti‐CCR2 antibody or isotype control from IR_D3 to IR_D6. Peripheral blood was collected at IR_D7, and cardiac tissues were collected at IR_D30. (B) Representative flow‐cytometry plots of circulating CCR2^+^Ly6C^hi^ monocytes in peripheral blood from isotype‐control and anti‐CCR2‐treated mice. (C) Quantification of circulating CCR2^+^Ly6C^hi^ monocytes among CD45^+^ leukocytes. Data are shown as box‐and‐whisker plots with individual data points. (D, E) Representative Masson's trichrome (D) and Sirius Red (E) staining of cardiac tissues from isotype‐control and anti‐CCR2‐treated mice. (F, G) Representative immunohistochemical staining of Collagen I (F) and α‐SMA (G). Boxed regions are shown at higher magnification on the right. (H–K) Quantification of Masson's trichrome‐positive area (H), Sirius Red‐positive area (I), Collagen I‐positive area (J), and α‐SMA‐positive area (K).  Scale bars, 50 µm. n = 6 mice per group. Data in panels H–K are presented as mean ± SEM. Statistical significance in panels C and H–K was assessed using an unpaired two‐tailed Student's t‐test. ****p < 0.0001.

At IR_D30, Masson's trichrome and Sirius Red staining showed reduced interstitial collagen deposition in anti‐CCR2‐treated mice (Figure 3D,E,H,I). A similar decrease in perivascular collagen deposition was observed with both stains (Figure S4A–D). Consistently, cardiac Collagen I and interstitial α‐SMA staining were also decreased after CCR2 neutralisation (Figure 3F,G,J,K). Quantification confirmed significant reductions in both interstitial and perivascular collagen deposition, together with smaller interstitial Collagen I‐positive and α‐SMA‐positive areas, compared with isotype controls (*p* < 0.0001). Together, these findings show that reduction of circulating CCR2^+^Ly6C^hi^ monocytes following CCR2 neutralisation is accompanied by attenuated cardiac fibrotic remodelling, supporting a contributory role for CCR2^+^ monocytes in radiation‐induced cardiac fibrosis.

### 3.4 AXL is upregulated in CCR2^+^ monocytes during radiation‐induced cardiac remodelling

To identify genes associated with the later phase of cardiac remodelling, we compared bulk cardiac transcriptomes from IR_D0, IR_D7, and IR_D30 mice. Differential‐expression analysis revealed distinct transcriptional changes across the three time points (Figure S5A–C). We then intersected the top 10 significantly upregulated genes, ranked by adjusted *p*‐value, from the IR_D30 versus IR_D0 and IR_D30 versus IR_D7 comparisons. This analysis identified four shared genes: Ccr2, Axl, Fn1, and Thbs1 (Figure 4A). The presence of Ccr2 was consistent with the monocyte accumulation identified above, whereas Fn1 and Thbs1 accompanied the fibrotic remodelling at IR_D30. We therefore examined the temporal and cellular expression of Axl, which encodes a receptor tyrosine kinase. Bulk RNA‐seq showed that Axl expression was higher at IR_D30 than at IR_D0 or IR_D7 (Figure 4B).

**FIGURE 4:**
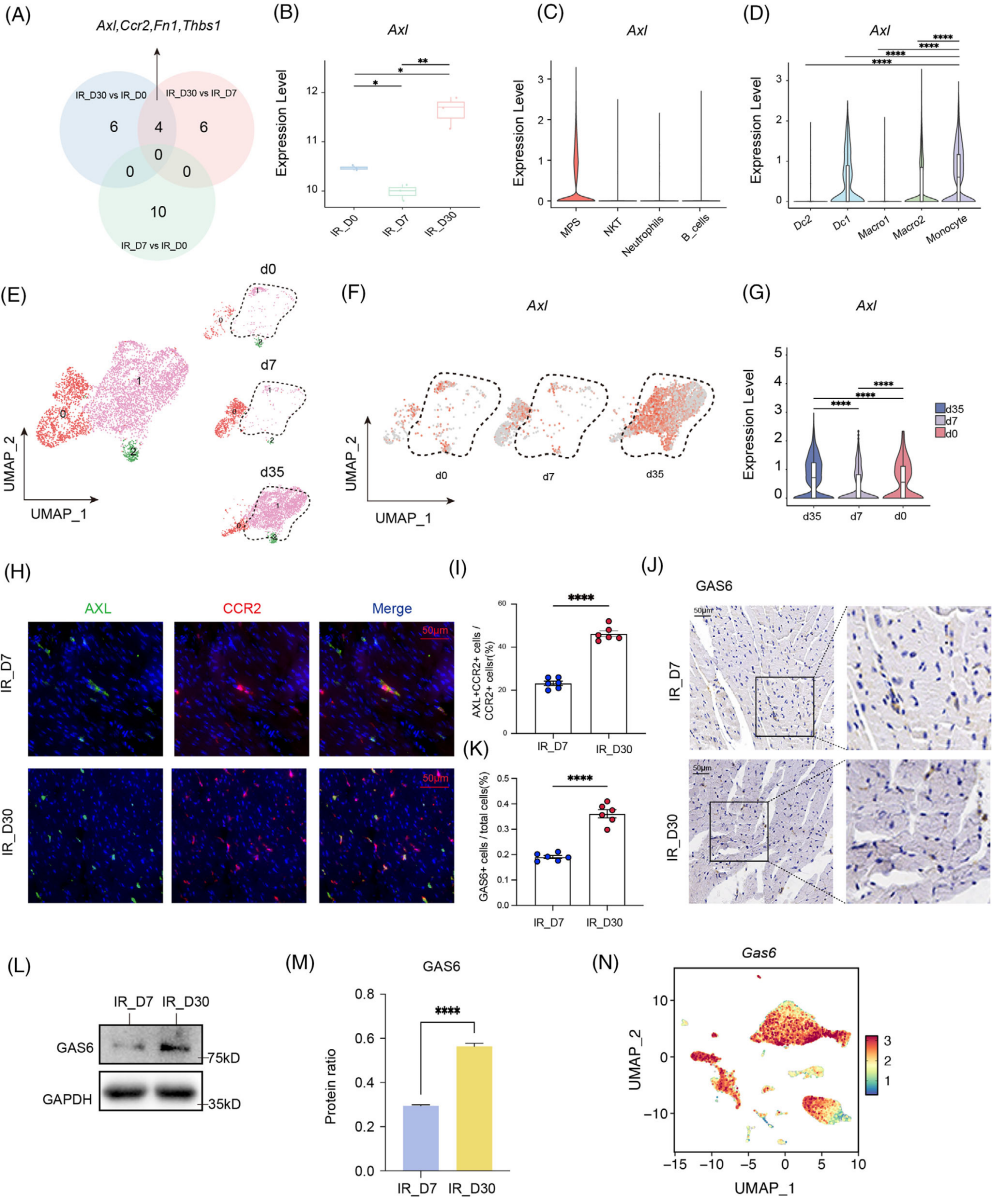
**AXL expression is enriched in cardiac monocytes during radiation‐induced cardiac remodeling**. (A) Venn diagram showing the overlap among the top 10 significantly upregulated genes, ranked by adjusted P value, from the IR_D30 versus IR_D0, IR_D30 versus IR_D7, and IR_D7 versus IR_D0 comparisons. (B) *Axl* expression in bulk cardiac RNA‐sequencing data from the IR_D0, IR_D7, and IR_D30 groups. (C) Violin plots showing *Axl* expression across the major cardiac immune‐cell populations, including the mononuclear phagocyte system (MPS), NKT cells, Neutrophils, and B cells, in the public radiation‐associated single‐cell RNA‐sequencing dataset (PRJNA1088304). (D) Violin plots showing *Axl* expression across Dc2, Dc1, Macro1, Macro2, and Monocyte populations within the MPS compartment. (E) UMAP visualization of monocyte subclusters and their distributions at d0, d7, and d35 in the public dataset. (F) Feature plots showing *Axl* expression in monocytes at d0, d7, and d35. (G) Violin plots showing monocyte Axl expression across the indicated time points. (H) Representative immunofluorescence staining of AXL and CCR2 in cardiac tissues at IR_D7 and IR_D30. Scale bar, 50 µm. n = 6 mice per group. (I) Quantification of the percentage of AXL^+^CCR2^+^ cells among CCR2^+^ cells. (J) Representative immunohistochemical staining of GAS6 in cardiac tissues at IR_D7 and IR_D30. Scale bar, 50 µm. n = 6 mice per group. (K) Quantification of GAS6‐positive cells in cardiac tissues at IR_D7 and IR_D30. (L, M) Western blotting (L) and densitometric quantification (M) of GAS6 protein expression in cardiac tissues at IR_D7 and IR_D30. n = 3 independent biological samples per group. Feature plot showing Gas6 expression across cardiac cell populations in the public cardiac remodeling and fibrosis single‐cell dataset (E‐MTAB‐8810). Data in panels I, K, and M are presented as mean ± SEM. Statistical significance in panel B was assessed using one‐way ANOVA followed by Tukey's multiple‐comparisons test, whereas panels I, K, and M were analyzed using an unpaired two‐tailed Student's *t*‐test. *p < 0.05; **p < 0.01; ****p < 0.0001.

Across the major cardiac immune‐cell populations in the radiation‐associated dataset, Axl expression was predominantly detected within the MPS compartment (Figure 4C). Further comparison of the five MPS populations showed the most prominent expression in monocytes (Figure 4D). Within the monocyte compartment, Axl expression was mapped across the three time points and was higher at d35 than at d0 or d7 (Figure 4E–G). In our irradiation model, immunofluorescence staining revealed a higher proportion of AXL^+^CCR2^+^ cells within the CCR2‐positive population at IR_D30 than at IR_D7 (Figure 4H,I). These observations confirmed that the increase in cardiac AXL expression was closely associated with CCR2‐positive monocytes during the later remodelling phase.

We next examined GAS6, a canonical ligand of AXL, to assess whether the cardiac tissue environment could support AXL activation. The proportion of GAS6‐positive cells was increased at IR_D30 compared with IR_D7 (Figure 4J,K), and western blotting confirmed a corresponding increase in cardiac GAS6 protein (Figure 4L, M). In a whole‐heart single‐cell reference dataset, Gas6 expression was detected mainly in fibroblasts and endothelial cells, with additional expression in a subset of myeloid cells (Figure 4N). Together, the IR_D30 remodelling phase was characterised by an increased abundance of AXL‐expressing CCR2‐positive monocytes and a GAS6‐enriched cardiac environment that may favour AXL activation in this population.

### 3.5 Myeloid Axl deletion and pharmacological AXL inhibition attenuate radiation‐induced cardiac fibrosis

To examine the contribution of myeloid AXL to cardiac fibrotic remodelling, we generated tamoxifen‐inducible myeloid‐specific Axl conditional‐knockout mice (Lyz2^CreERT2^; Axl^fl/fl^; Rosa26^tdTomato^; Figure 5A). Control and Axl‐cKO mice received whole‐thorax irradiation, and cardiac tissues were collected at IR_D30 (Figure 5B). In flow‐sorted cardiac CCR2^+^Ly6C^hi^ monocytes, AXL protein was readily detected in Control mice but was undetectable after Axl deletion (Figure 5C). Immunofluorescence analysis further showed a pronounced loss of AXL signal in RFP‐labelled myeloid‐lineage cells from Axl‐cKO hearts (Figure 5D).

**FIGURE 5:**
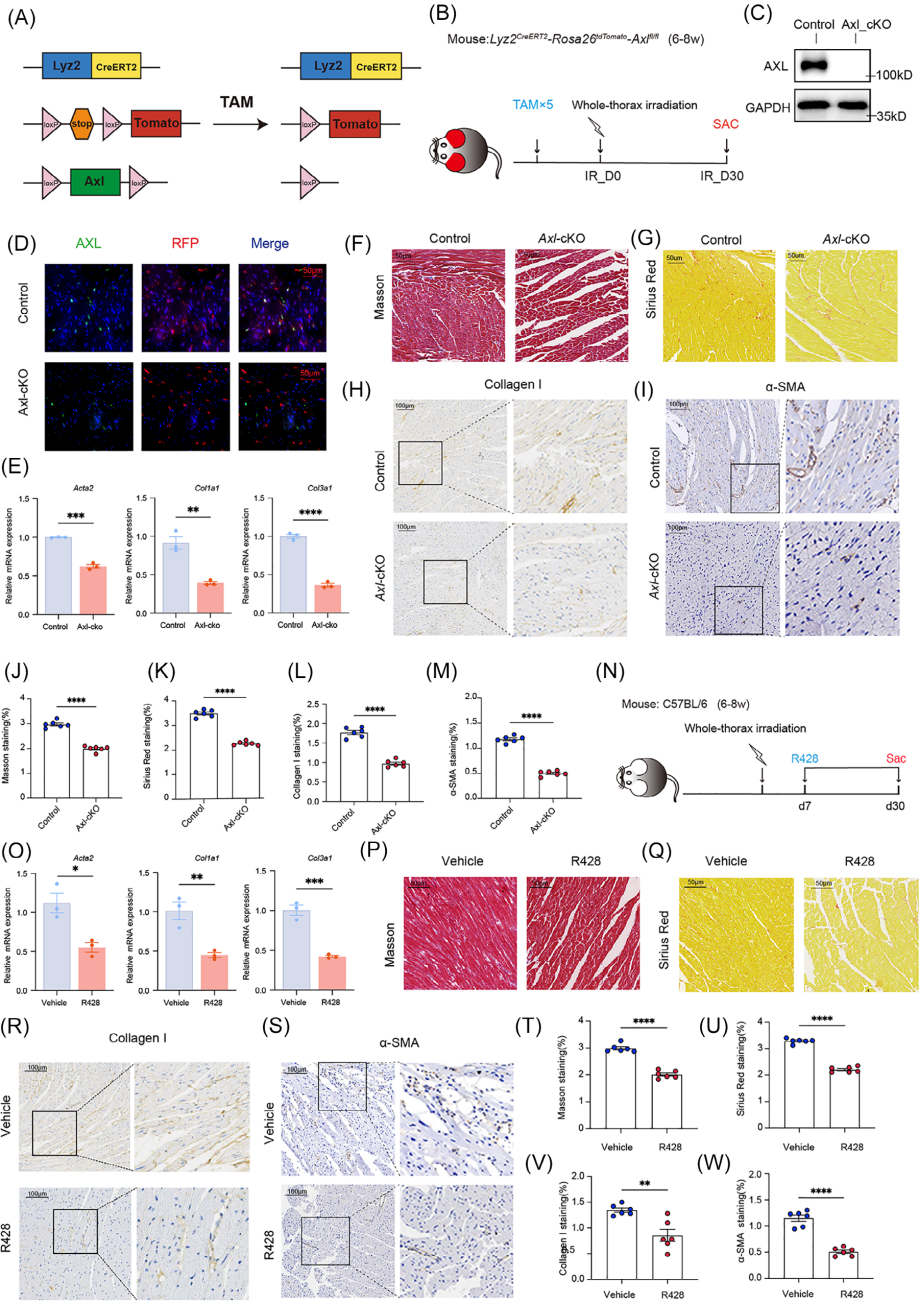
**Myeloid Axl deletion and pharmacological AXL inhibition attenuate radiation‐induced cardiac fibrosis**. (A) Schematic diagram of the generation of tamoxifen‐inducible myeloid Axl conditional‐knockout mice. (B) Schematic diagram of the experimental design. Control and Axl‐cKO mice underwent whole‐thorax irradiation, and cardiac tissues were collected at IR_D30. (C) Representative western blot analysis of AXL protein in flow‐sorted cardiac CCR2^+^Ly6C^hi^ monocytes from Control and Axl‐cKO mice at IR_D30. GAPDH served as the loading control. (D) Representative immunofluorescence staining of AXL and RFP in cardiac tissues from Control and Axl‐cKO mice at IR_D30. Green, AXL; red, RFP; blue, DAPI. (E) Relative mRNA expression of *Acta2*, *Col1a1*, and *Col3a1* in cardiac tissues from Control and Axl‐cKO mice at IR_D30, as determined by quantitative real‐time PCR (qPCR). (F, G) Representative Masson's trichrome (F) and Sirius Red (G) staining of cardiac tissues from Control and Axl‐cKO mice at IR_D30. (H, I) Representative immunohistochemical staining of Collagen I (H) and α‐SMA (I) in cardiac tissues from Control and Axl‐cKO mice. Boxed regions are shown at higher magnification on the right. (JM) Quantification of the Masson's trichrome‐positive area (J), Sirius Red‐positive area (K), Collagen I‐positive area (L), and α‐SMA‐positive area (M), expressed as percentages of the analyzed myocardial interstitial area. (N) Schematic diagram of the R428 treatment schedule following whole‐thorax irradiation. Mice received R428 (25 mg/kg) or vehicle by oral gavage once daily from IR_D7 to IR_D30. Cardiac tissues were collected at IR_D30. (O) Relative mRNA expression of *Acta2*, *Col1a1*, and *Col3a1* in cardiac tissues from Vehicle‐ and R428‐treated mice at IR_D30, as determined by qPCR. (P, Q) Representative Masson's trichrome (P) and Sirius Red (Q) staining of cardiac tissues from Vehicle‐ and R428‐treated mice at IR_D30. (R, S) Representative immunohistochemical staining of Collagen I (R) and α‐SMA (S) in cardiac tissues from Vehicle‐ and R428‐treated mice. Boxed regions are shown at higher magnification on the right. (TW) Quantification of the Masson's trichrome‐positive area (T), Sirius Red‐positive area (U), Collagen I‐positive area (V), and α‐SMA‐positive area (W), expressed as percentages of the analyzed myocardial interstitial area. Scale bars, 50 µm in D, F, G, P, and Q; 100 µm in the left‐hand images in H, I, R, and S. n = 6 mice per group for histological analyses and n = 3 independent biological samples per group for qPCR analyses. For sorted‐monocyte immunoblotting in C, n = 3 independent pooled biological replicates, with cells from 23 mice pooled for each replicate. Quantitative data are presented as mean ± SEM. Statistical significance was assessed using an unpaired two‐tailed Student's *t*‐test. *p < 0.05; **p < 0.01; ***p < 0.001; ****p < 0.0001.

At IR_D30, quantitative real‐time PCR (qPCR) showed significantly lower *Acta2*, *Col1a1*, and *Col3a1* mRNA levels in Axl‐cKO hearts than in Control hearts (Figure 5E). Consistent with these molecular changes, Masson's trichrome and Sirius Red staining revealed reduced interstitial collagen deposition (Figure 5F,G,J,K), with a similar decrease in perivascular regions (Figure S6A–D). Immunohistochemical analysis further showed smaller Collagen I‐positive and α‐SMA‐positive areas in the myocardial interstitium (Figure 5H,I,L,M). Myeloid Axl deletion therefore limited collagen accumulation and myofibroblast‐associated activation following irradiation.

As an independent pharmacological approach, irradiated mice received the AXL inhibitor bemcentinib (R428) from IR_D7 to IR_D30, while control mice received vehicle (Figure 5N). Similar to the findings in Axl‐cKO hearts, R428 treatment significantly reduced cardiac Acta2, Col1a1, and Col3a1 mRNA levels (Figure 5O). At the tissue level, both Masson's trichrome and Sirius Red staining showed less collagen deposition in the myocardial interstitium (Figure 5P,Q,T,U) and perivascular regions (Figure S6E–H). These changes were accompanied by reduced interstitial Collagen I and α‐SMA staining (Figure 5R,S,V,W). Thus, systemic AXL blockade reproduced the antifibrotic phenotype observed after myeloid Axl deletion.

Together, these findings identify myeloid AXL as a functional contributor to radiation‐induced cardiac fibrosis and highlight the therapeutic potential of AXL inhibition.

### 3.6 Genetic and pharmacological AXL targeting dampens MAPK signalling in cardiac CCR2^+^Ly6C^hi^ monocytes after irradiation

To define the transcriptional changes following myeloid Axl deletion, we compared cardiac transcriptomes from irradiated Control and Axl‐cKO mice (Figure 6A). Volcano analysis showed reduced *Axl* and *Mertk* expression in Axl‐cKO hearts. Other differentially expressed genes included *Map2k1*, *Map2k2*, and *Dusp9* (Figure 6B).

**FIGURE 6:**
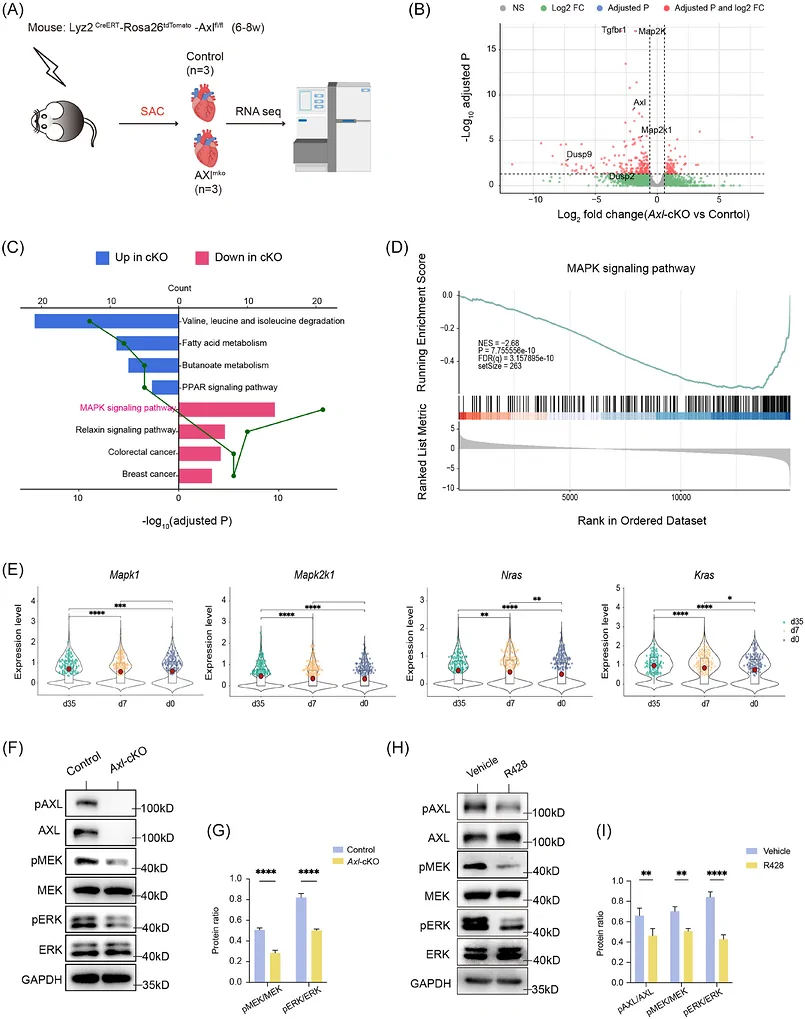
**AXL regulates MEK/ERK‐MAPK signaling in cardiac CCR2^+^Ly6C^hi^ monocytes**. (A) Schematic diagram of bulk RNA‐sequencing analysis of irradiated Control and Axl‐cKO hearts (n = 3 independent biological samples per group). (B) Volcano plot showing differentially expressed genes between Axl‐cKO and Control groups. (C) KEGG pathway‐enrichment analysis of upregulated and downregulated genes in Axl‐cKO hearts. (D) GSEA showing negative enrichment of the MAPK signaling gene set in Axl‐cKO versus Control cardiac transcriptomes. (E) Violin plots showing *Mapk1*, *Map2k1*, *Nras*, and *Kras* expression in monocytes across different time points in the cardiac single‐cell dataset. (F) Western blot analysis of pAXL, AXL, pMEK, MEK, pERK, ERK, and GAPDH in flow‐sorted cardiac CCR2+Ly6Chi monocytes from Control and Axl‐cKO mice. (G) Quantification of pMEK/MEK and pERK/ERK protein levels in panel F. (H) Western blot analysis of pAXL, AXL, pMEK, MEK, pERK, ERK and GAPDH in cardiac CCR2+Ly6Chi monocytes isolated at IR_D30 from irradiated mice treated systemically with vehicle or R428. (I) Quantification of pAXL/AXL, pMEK/MEK, and pERK/ERK protein levels in panel H. GAPDH served as the loading control. For monocyte immunoblotting in FI, n = 3 independent pooled biological replicates per group, with cells from 23 mice pooled for each replicate. Data in G and I are presented as mean ± SEM. Statistical significance for these comparisons was assessed using an unpaired two‐tailed Student's *t*‐test. *p < 0.05; **p < 0.01; ****p < 0.0001.

KEGG analysis was subsequently performed on the differentially expressed genes. MAPK signalling ranked as the top enriched pathway among genes downregulated in Axl‐cKO hearts (Figure 6C). GSEA likewise showed negative enrichment of the same pathway, consistent with an overall decrease in MAPK‐related gene expression following myeloid Axl deletion (Figure 6D). In the radiation‐associated cardiac single‐cell dataset, monocyte expression of *Mapk1*, *Map2k1*, *Nras*, and *Kras* also varied across d0, d7 and d35 (Figure 6E).

These transcriptomic findings prompted us to examine MEK/ERK activity in flow‐sorted cardiac CCR2^+^Ly6Cʰ^i^ monocytes. Myeloid Axl deletion was associated with lower pMEK/MEK and pERK/ERK ratios, without comparable changes in total MEK or ERK abundance (Figure 6F,G). A similar pattern was observed after pharmacological AXL inhibition: monocytes isolated from irradiated mice treated systemically with R428 showed lower pAXL/AXL, pMEK/MEK and pERK/ERK ratios than those from vehicle‐treated mice (Figure 6H,I). Together, these findings link AXL targeting to reduced MEK/ERK phosphorylation in cardiac CCR2^+^Ly6Cʰ^i^ monocytes after irradiation.

### 3.7 AXL‐MEK/ERK signalling in cardiac CCR2^+^Ly6C^hi^ monocytes promotes paracrine fibroblast activation

To determine whether cardiac CCR2^+^Ly6C^hi^ monocytes communicate with fibroblasts through soluble factors, we established a conditioned‐medium system using flow‐sorted monocytes and primary cardiac fibroblasts (Figure 7A). Medium collected from IR_D30 monocytes increased COL1A1 and α‐SMA expression in fibroblasts compared with medium from Sham monocytes (Figure 7B,C). This fibroblast‐activating effect was markedly weakened when the donor cells were obtained from A*xl*‐cKO mice (Figure 7D,E), indicating that monocyte AXL contributes to the profibrotic paracrine activity acquired after irradiation.

**FIGURE 7:**
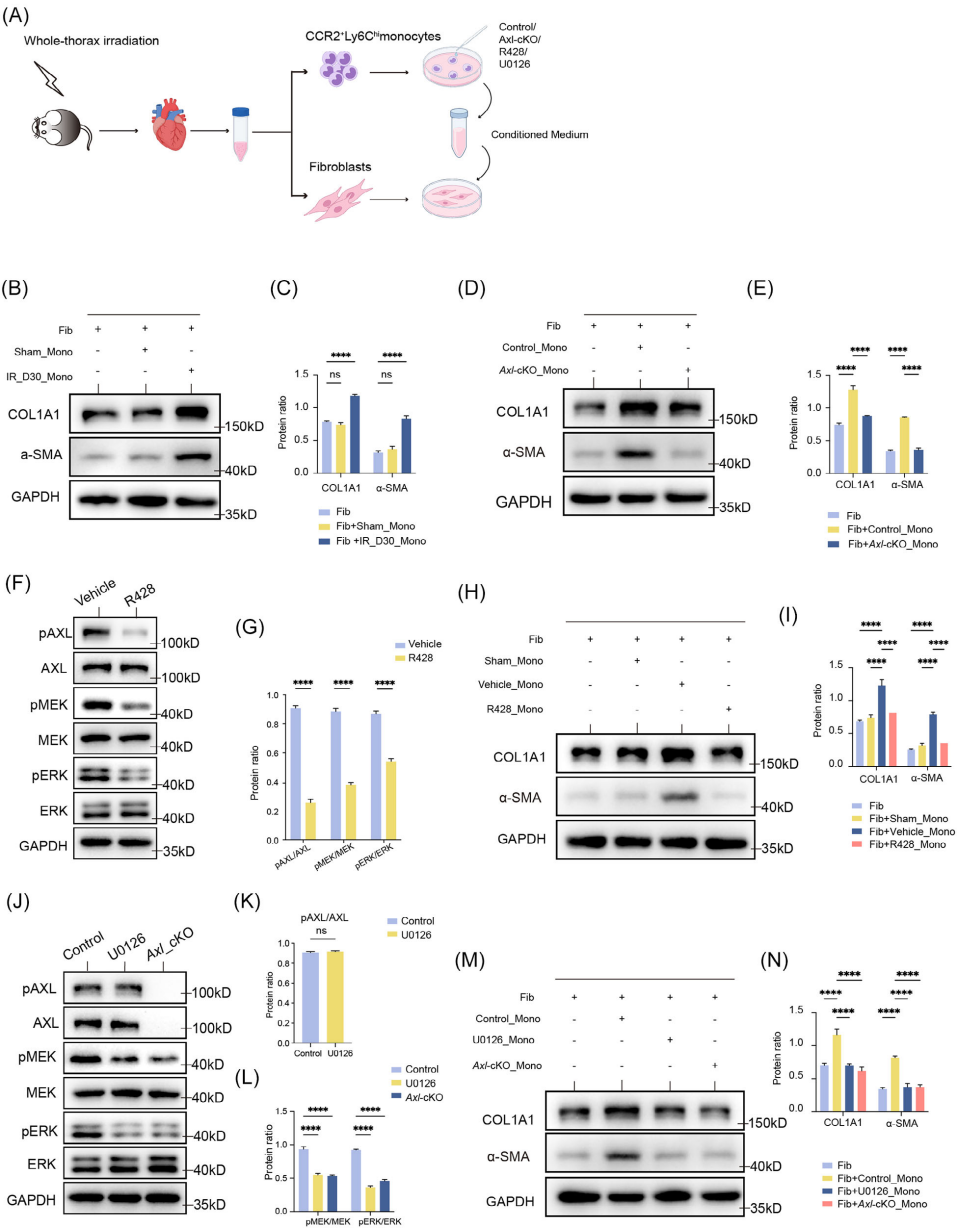
**AXL–MEK/ERK signaling in cardiac CCR2^+^Ly6C^hi^ monocytes promotes fibroblast activation**. (A) Schematic diagram of the monocyte‐conditioned medium experiments. Cardiac CCR2^+^Ly6C^hi^ monocytes were isolated from Sham or irradiated hearts under the indicated genetic conditions or treated ex vivo with inhibitors, and the collected conditioned medium was applied to primary cardiac fibroblasts. (B) Western blotting of COL1A1, α‐SMA, and GAPDH in cardiac fibroblasts cultured alone or with conditioned medium from Sham or IR_D30 monocytes. (C) Quantification of COL1A1 and α‐SMA protein levels in panel B. (D) Western blotting of COL1A1, α‐SMA, and GAPDH in cardiac fibroblasts cultured alone or with conditioned medium from Control or Axl‐cKO monocytes. (E) Quantification of COL1A1 and α‐SMA protein levels in panel D. (F) Western blotting of pAXL, AXL, pMEK, MEK, pERK, ERK, and GAPDH in flow‐sorted IR_D30 cardiac CCR2^+^Ly6C^hi^ monocytes treated ex vivo with vehicle or R428. (G) Quantification of pAXL/AXL, pMEK/MEK and pERK/ERK protein levels in panel F. (H) Western blotting of COL1A1, α‐SMA, and GAPDH in cardiac fibroblasts cultured alone or with conditioned medium from Sham monocytes or IR_D30 monocytes treated with vehicle or R428. (I) Quantification of COL1A1 and α‐SMA protein levels in panel H. (J) Western blotting of pAXL, AXL, pMEK, MEK, pERK, ERK, and GAPDH in Control, U0126‐treated, and Axl‐cKO cardiac CCR2^+^Ly6C^hi^ monocytes. (K) Quantification of pAXL/AXL protein levels in Control and U0126‐treated monocytes. (L) Quantification of pMEK/MEK and pERK/ERK protein levels in Control, U0126‐treated, and Axl‐cKO monocytes. (M) Western blotting of COL1A1, α‐SMA, and GAPDH in cardiac fibroblasts cultured alone or with conditioned medium from Control, U0126‐treated, or Axl‐cKO monocytes. (N) Quantification of COL1A1 and α‐SMA protein levels in panel M. For sorted‐monocyte immunoblotting in F, G, and J–L, n = 3 independent pooled biological replicates per condition, with cells from 23 mice pooled for each replicate. For the fibroblast conditioned‐medium experiments quantified in C, E, I, and N, n = 3 independent biological replicates per condition. Data are presented as mean ± SEM. Panels G and K were analyzed using an unpaired two‐tailed Student's *t*‐test, whereas panels C, E, I, L, and N were analyzed using one‐way ANOVA followed by Tukey's multiple‐comparisons test. ns, not significant; ****p < 0.0001.

We further examined this effect by directly inhibiting AXL in isolated IR_D30 monocytes. R428 reduced AXL phosphorylation and concurrently lowered MEK and ERK phosphorylation (Figure 7F,G). Fibroblasts exposed to medium from R428‐treated monocytes expressed less COL1A1 and α‐SMA than those receiving medium from Vehicle‐treated cells (Figure 7H,I). Direct AXL inhibition within the monocyte culture was therefore sufficient to weaken its profibrotic paracrine activity.

The functional contribution of MEK/ERK signalling was assessed using the MEK1/2 inhibitor U0126. U0126 treatment reduced MEK and ERK phosphorylation, whereas no significant difference in the pAXL/AXL ratio was detected between Control and U0126‐treated monocytes (Figure 7J–L). Medium derived from these cells elicited substantially lower COL1A1 and α‐SMA expression in cardiac fibroblasts, consistent with the attenuated response observed with Axl‐cKO monocyte medium (Figure 7M,N). These findings identify MEK/ERK signalling as part of the AXL‐dependent paracrine program through which cardiac CCR2^+^Ly6C^hi^ monocytes promote fibroblast activation after irradiation.

## 4 DISCUSSION

This study identifies a transitional stage of radiation‐induced cardiac remodelling in which inflammatory changes remain detectable after fibrosis has become established and links this tissue state to increased AXL expression within CCR2^+^ monocytes. The principal advance is not the association of CCR2^+^ monocytes or AXL with fibrosis in isolation, as both have been implicated in other forms of tissue injury. Rather, our data connect AXL activity in this cardiac monocyte population to MEK/ERK signalling and to a paracrine program that promotes fibroblast activation after irradiation. Evidence from CCR2 neutralisation, inducible myeloid *Axl* deletion, pharmacological AXL inhibition, signalling analyses in sorted monocytes, and conditioned‐medium experiments supports this relationship across tissue, cellular, and molecular levels. This framework places monocyte AXL signalling at the interface between radiation‐associated immune remodelling and the developing fibrotic niche.

The temporal context is important for interpreting this mechanism. Radiation‐induced cardiac injury is often described as a progression from acute cellular damage and inflammation to extracellular matrix accumulation and chronic remodeling.^4^, ^5^, ^34^ Our observations indicate that these processes are not strictly sequential. At IR_D30, collagen deposition and myofibroblast‐associated staining were already evident, whereas leukocyte infiltration and apoptotic signals, although lower than at IR_D7, remained above baseline. IR_D30 therefore marks a period in which inflammatory and immune‐cell changes coexist with an established fibrotic environment. Consistent with this interpretation, whole‐heart deconvolution suggested concurrent remodelling of both immune and stromal compartments, with a higher estimated fibroblast proportion at IR_D30. This stromal change was concordant with the increased collagen deposition and myofibroblast‐associated staining observed histologically. Together, these findings support the view that immune remodelling at this stage occurs in parallel with an expanding fibrotic stromal compartment rather than as an isolated inflammatory response. This interpretation is also consistent with single‐cell evidence that cardiac macrophage states continue to evolve during radiation injury.^13^

CCR2^+^ monocytes provide one cellular link between these overlapping responses. In other forms of cardiac injury, CCR2^+^ monocyte influx supplements resident macrophage populations and promotes adverse remodelling through inflammatory and profibrotic programs.^17^, ^18^ Radiation‐associated immune single‐cell analysis identified late changes within the mononuclear phagocyte compartment, while lineage tracing showed increased cardiac accumulation of CCR2^+^RFP^+^ cells at IR_D30 compared with IR_D7. Peripheral blood flow cytometry further confirmed that anti‐CCR2 treatment markedly reduced circulating CCR2^+^Ly6C^hi^ monocytes. This reduction was accompanied by decreased interstitial collagen deposition and lower fibrotic marker expression in irradiated hearts, providing functional support for the contribution of CCR2^+^ monocytes to radiation‐induced cardiac fibrotic remodelling. These findings extend previous descriptions of radiation‐associated macrophage heterogeneity by identifying CCR2^+^ monocytes as a functionally relevant immune population during cardiac fibrosis.^13^ Other infiltrating or resident immune populations may also contribute to remodelling,^10^, ^11^ but the present data support a tractable cellular route through which the immune response can influence fibrotic remodelling.

Within this monocyte population, AXL emerged as a candidate regulator of profibrotic activity. TAM receptors regulate immune‐cell survival, efferocytosis, migration, and adaptation to tissue injury, and their effects vary with cell state and disease context.^20^ Cardiac AXL expression is elevated in human heart failure and during the early phase of pressure‐overload remodelling, linking this receptor to adverse cardiac remodelling beyond radiation injury.^23^, ^24^ Macrophage AXL also promotes inflammatory metabolic reprogramming after myocardial reperfusion.^25^ In the lung, AXL inhibition has been shown to attenuate profibrotic monocyte‐derived macrophage responses and pulmonary fibrosis.^22^ However, the effects of AXL inhibition can differ with the stage of injury. In a separate bleomycin‐induced lung injury model, inhibition during the acute phase exacerbated inflammation and subsequent fibrosis, whereas inhibition during the fibrotic phase reduced fibrosis.^35^ These studies establish the broader relevance of AXL but do not define an immune‐cell‐specific route from cardiac AXL signalling to fibroblast activation. Here, the increased proportion of AXL‐positive cells within the CCR2^+^ compartment, together with the attenuation of fibrosis after myeloid Axl deletion, supports a contribution of AXL signalling in CCR2^+^ monocytes to fibrotic remodelling in the irradiated heart. The accompanying increase in cardiac GAS6 suggests a GAS6‐enriched microenvironment that may support AXL activation at this stage, without establishing GAS6 as the cause of *Axl* induction or implying direct regulation between CCR2 and AXL.

Transcriptomic analyses of irradiated control and myeloid Axl‐deficient hearts implicated MAPK‐related programs associated with Axl loss, prompting experimental testing of the MEK/ERK branch. In sorted cardiac CCR2^+^Ly6C^hi^ monocytes, both myeloid Axl deletion and AXL inhibition reduced MEK and ERK phosphorylation without comparable changes in total MEK or ERK abundance. Thus, AXL is linked to MEK/ERK pathway activity at the phosphorylation level. This relationship accords with established coupling of TAM receptors to GRB2 and downstream MEK/ERK signalling.^20^ MEK1/2 inhibition with U0126 also weakened the fibroblast‐activating effect of monocyte‐CM, resembling the effect of *Axl* deletion. These data place MEK/ERK within the AXL‐dependent monocyte response, while leaving open the contribution of other MAPK branches and additional AXL effectors.

The conditioned‐medium experiments reveal how this signalling state can influence the fibrotic niche. Fibroblasts are the principal matrix‐producing cells in cardiac fibrosis, and the higher estimated fibroblast representation at IR_D30, together with the histological evidence of matrix accumulation, supports active stromal remodelling at this stage.^7^, ^8^ Importantly, fibroblast activation is shaped by signals from the surrounding immune microenvironment.^9^, ^36^ Previous studies in heart failure and experimental cardiac injury have identified extensive communication between CCR2^+^ monocyte/macrophage populations and activated fibroblasts.^12^, ^14^ Our findings extend this concept to radiation injury alone by identifying AXL–MEK/ERK signalling as a regulator, at least in part, of the fibroblast‐activating activity of cardiac CCR2^+^Ly6C^hi^ monocytes. Conditioned medium from IR_D30 monocytes increased COL1A1 and α‐SMA expression in primary cardiac fibroblasts, whereas *Axl* deletion, direct AXL inhibition, or MEK1/2 inhibition in the donor monocytes diminished this response. These findings establish an AXL‐dependent paracrine effect on fibroblast activation, while the soluble mediators responsible for this response remain to be defined.

Several inflammatory and profibrotic factors have emerged as plausible mediators of monocyte/macrophage‐driven fibroblast activation. In myocardial ischemia and reperfusion injury, macrophage AXL has been shown to promote IL‐1β production.^25^ More recently, studies in heart failure demonstrated that IL‐1β derived from CCR2^+^ monocytes/macrophages directly promotes the emergence of activated fibroblast states and contributes to myocardial fibrosis.^12^ Other macrophage‐derived mediators, including PDGF, osteopontin/SPP1, amphiregulin, and TGF‐β, have also been implicated in fibroblast activation and extracellular matrix remodelling in different forms of cardiac injury.^10^ Among these mediators, TGF‐β1 promotes myofibroblast differentiation and matrix production through canonical SMAD signalling, but its effects are not restricted to this pathway. TGF‐β receptors can also engage SHCA and the GRB2–SOS complex to activate the RAS–RAF–MEK–ERK cascade, linking TGF‐β signalling to a pathway also activated by receptor tyrosine kinases such as AXL.^37^ In addition to this overlap in downstream signalling, TGF‐β1 can regulate AXL expression. In a study of inflammatory arthritis, TGF‐β1 increased AXL expression in human macrophages and synovial micromasses, while local TGF‐β1 overexpression also induced synovial AXL expression in mice.^38^ Whether these interactions also influence the fibroblast‐activating activity of irradiated cardiac monocytes remains unclear. Multiple paracrine mediators may cooperate to influence fibroblast activation and matrix remodelling, and identifying the key effectors and their regulation by AXL–MEK/ERK signalling remains an important direction for future studies of radiation‐induced cardiac fibrosis.

Several limitations should be considered. First, the inducible Lyz2^CreERT2^ model targets the broader myeloid lineage rather than monocytes exclusively, although CCR2 neutralisation and experiments using sorted CCR2^+^Ly6C^hi^ monocytes provide complementary evidence supporting the involvement of this population. Second, the public radiation‐associated single‐cell dataset contained d0, d7, and d35 samples, making d35 a supportive late‐stage reference rather than an exact match for IR_D30, while the E‐MTAB‐8810 reference was derived from an AngII model and therefore provides cross‐model estimates of cardiac cell composition. Third, the single‐dose 20 Gy whole‐thorax irradiation model differs from the fractionated exposure used clinically and may not fully reproduce the kinetics of human radiotherapy‐associated cardiac injury. Finally, cardiac functional outcomes and human radiotherapy‐associated cardiac specimens were not assessed; therefore, reduced histological fibrosis cannot be equated with improved cardiac function, and the clinical relevance of the CCR2^+^ monocyte–AXL axis remains to be established.

Together, these findings indicate that AXL signalling in cardiac CCR2^+^ monocytes promotes paracrine fibroblast activation, at least in part through MEK/ERK, thereby contributing to radiation‐induced cardiac fibrosis.

## 5 CONCLUSIONS

In conclusion, IR_D30 represents a stage characterised by persistent immune‐cell alterations within an established fibrotic cardiac environment. In this setting, AXL signalling in cardiac CCR2^+^ monocytes contributes to their capacity to promote fibroblast activation, at least in part through MEK/ERK signalling. These findings link irradiation‐induced changes in cardiac CCR2^+^ monocytes to fibroblast activation and support further evaluation of AXL as a potential therapeutic target for radiation‐induced cardiac fibrosis.

## AUTHOR CONTRIBUTIONS

  .

## CONFLCITS OF INTEREST STATEMENT

The authors declare no conflict of interest.

## Supporting information



Supporting Information

## Data Availability

The datasets used and/or analyzed during the current study are available from the corresponding authors upon reasonable request. All materials related to the study can also be requested.
