# Supplementary material for: Radiation‐induced AXL upregulation in CCR2+ monocytes promotes cardiac fibrosis
Source: Clin Transl Med. 2026 Oct 13;16(10):e70852. doi: 10.1002/ctm2.70852 (PMC13659278; doi:10.1002/ctm2.70852)
Supplement: Supplementary file 1 [file CTM2-16-e70852-s001.docx]

**Figure S1. Temporal changes in perivascular collagen deposition following whole-thorax irradiation.**

(A) Representative Masson’s trichrome staining of cardiac perivascular regions in the Sham, IR_D7, IR_D30, and IR_D60 groups.

(B) Quantification of the Masson’s trichrome-positive area in perivascular regions.

(C) Representative Sirius Red staining of cardiac perivascular regions in the Sham, IR_D7, IR_D30, and IR_D60 groups.

(D) Quantification of the Sirius Red-positive area in perivascular regions.

Scale bars, 50 μm. Data are presented as mean ± SEM. n = 6 mice per group. Statistical significance was assessed using one-way ANOVA followed by Tukey’s multiple-comparisons test. ns, not significant; ****P < 0.0001.

****Figure S2. Cell-type annotation of the whole-heart single-cell/single-nucleus transcriptomic dataset (E-MTAB-8810).****
(A) UMAP visualization of the major cardiac cell populations identified in the whole-heart single-cell/single-nucleus transcriptomic dataset.
(B) Dot plot showing the expression of representative marker genes used to annotate the major cardiac cell populations.

**Figure S3. Single-cell characterization and annotation of cardiac immune-cell populations in the radiation-associated dataset (PRJNA1088304).**
(A) UMAP visualization of the major immune-cell populations, including MPS, NKT cells, Neutrophils, and B cells.
(B) Feature plots showing the expression of representative marker genes used to annotate MPS, B cells, Neutrophils, and NKT cells.
(C) Heatmap showing selected marker-gene expression across Dc2, Monocyte, Macro2, Macro1, and Dc1 populations.

**Figure S4. Anti-CCR2 treatment reduces perivascular collagen deposition in irradiated hearts.**

(A) Representative Masson’s trichrome staining of cardiac perivascular regions in isotype-control and anti-CCR2-treated mice at IR_D30.

(B) Quantification of the Masson’s trichrome-positive area in perivascular regions.

(C) Representative Sirius Red staining of cardiac perivascular regions in isotype-control and anti-CCR2-treated mice at IR_D30.

(D) Quantification of the Sirius Red-positive area in perivascular regions.

Scale bars, 50 μm in A and C. Data in panels B and D are presented as mean ± SEM; n = 6 mice per group. Statistical significance was assessed using an unpaired two-tailed Student’s t-test. ****P < 0.0001.

**Figure S5. Differential expression analysis of cardiac transcriptomes following irradiation.**
(A–C) Volcano plots showing differentially expressed genes in the IR_D7 versus IR_D0 (A), IR_D30 versus IR_D0 (B), and IR_D30 versus IR_D7 (C) comparisons.

Dashed lines indicate the thresholds of |log_2_ fold change| = 1 and adjusted P value = 0.05.

**Figure S6. Myeloid Axl deletion and pharmacological AXL inhibition reduce perivascular collagen deposition in irradiated hearts.**

(A) Representative Masson’s trichrome staining of cardiac perivascular regions in Control and *Axl*-cKO mice at IR_D30.

(B) Quantification of the Masson’s trichrome-positive area in perivascular regions from the groups shown in A.

(C) Representative Sirius Red staining of cardiac perivascular regions in Control and *Axl*-cKO mice at IR_D30.

(D) Quantification of the Sirius Red-positive area in perivascular regions from the groups shown in C.

(E) Representative Masson’s trichrome staining of cardiac perivascular regions in Vehicle- and R428-treated mice at IR_D30.

(F) Quantification of the Masson’s trichrome-positive area in perivascular regions from the groups shown in E.

(G) Representative Sirius Red staining of cardiac perivascular regions in Vehicle- and R428-treated mice at IR_D30.

(H) Quantification of the Sirius Red-positive area in perivascular regions from the groups shown in G.

Scale bars, 50 μm. Data are presented as mean ± SEM; n = 6 mice per group. Statistical significance was assessed using an unpaired two-tailed Student’s t-test. ****P < 0.0001.
